# Veteran Status Matters! Life Course Perspectives on the Health and Well-Being of Aging Veterans

**DOI:** 10.1093/geroni/igaa057.2179

**Published:** 2020-12-16

**Authors:** Avron Spiro

## Abstract

Military service during early life can result in exposure to traumatic events that can reverberate throughout life. Although much attention is focused on the negative effects of military service, many veterans report positive effects. These papers explore life course effects of military service on veterans’ health and well-being. Three used national US longitudinal cohorts (HRS, MIDUS); two sampled veterans from Oregon or from Korea. Three compared veterans to non-veterans; two examined veterans only. Cheng and colleagues found that veterans in HRS are more likely to be risk-averse than non-veterans. Risk aversion matters because it determines how people make decisions and predicts a wide array of health and economic outcomes. Kurth and colleagues examined Oregon veterans from several wars, finding PTSD symptoms were highest among Vietnam combat veterans, the oldest cohort; there were no differences among non-combat veterans. Piazza and colleagues examined in MIDUS the impact of veteran status on cortisol, a stress biomarker, finding older veterans more likely had non-normative patterns than did younger or non-veterans. Lee and colleagues studied patterns of mental health among Korean Vietnam veterans, identifying two patterns as ‘normal’ and ‘resilient’ encompassing half the sample; these veterans demonstrated positive outcomes of military service. Frochen and colleagues compared depression trajectories between veterans and non-veterans in HRS, finding veterans had less depression than non-veterans, but among veterans, trajectories varied based on extent of service. in sum, these papers demonstrate that military service can have positive as well as negative effects on veterans’ health and well-being in later life. Aging Veterans: Effects of Military Service across the Life Course Interest Group Sponsored Symposium.

